# Involvement and repair of epithelial barrier dysfunction in allergic diseases

**DOI:** 10.3389/fimmu.2024.1348272

**Published:** 2024-02-01

**Authors:** Hui-Fei Lu, Yi-Chi Zhou, Li-Tao Yang, Qian Zhou, Xi-Jia Wang, Shu-Qi Qiu, Bao-Hui Cheng, Xian-Hai Zeng

**Affiliations:** ^1^ Department of Graduate and Scientific Research, Zhuhai Campus of Zunyi Medical University, Zhuhai, China; ^2^ Department of Otolaryngology, Longgang Otolaryngology Hospital & Shenzhen Key Laboratory of Otolaryngology, Institute of Otolaryngology Shenzhen, Shenzhen, China; ^3^ Department of Gastroenterology, Beijing University of Chinese Medicine Shenzhen Hospital (Longgang), Shenzhen, China; ^4^ Clinical Laboratory Department of The Second Affiliated Hospital, School of Medicine, The Chinese University of Hong Kong, Shenzhen & Longgang District People’s Hospital of Shenzhen, Shenzhen, China

**Keywords:** epithelial barrier, allergic diseases, barrier dysfunction, type 2 inflammation, prevention and treatment

## Abstract

The epithelial barrier serves as a critical defense mechanism separating the human body from the external environment, fulfilling both physical and immune functions. This barrier plays a pivotal role in shielding the body from environmental risk factors such as allergens, pathogens, and pollutants. However, since the 19th century, the escalating threats posed by environmental pollution, global warming, heightened usage of industrial chemical products, and alterations in biodiversity have contributed to a noteworthy surge in allergic disease incidences. Notably, allergic diseases frequently exhibit dysfunction in the epithelial barrier. The proposed epithelial barrier hypothesis introduces a novel avenue for the prevention and treatment of allergic diseases. Despite increased attention to the role of barrier dysfunction in allergic disease development, numerous questions persist regarding the mechanisms underlying the disruption of normal barrier function. Consequently, this review aims to provide a comprehensive overview of the epithelial barrier’s role in allergic diseases, encompassing influencing factors, assessment techniques, and repair methodologies. By doing so, it seeks to present innovative strategies for the prevention and treatment of allergic diseases.

## 1 Introduction

Allergic diseases, characterized by inflammatory responses mediated by type 2 immune reactions, encompass conditions such as asthma, atopic rhinitis (AR), atopic dermatitis (AD), and food allergy (FA), arising from the intricate interplay of genetic and environmental factors (1). Since the 19th century, the intensification of industrialization and modernization has contributed to escalating environmental pollution, particularly in urban areas, exerting a pronounced impact on human health (2). Current research unequivocally affirms the pivotal role of environmental factors in the initiation and progression of allergic diseases. The adverse effects of industrialization, urbanization, and modernization on the environment, spanning air pollution, global warming, climate alterations, and biodiversity depletion, have led to a marked surge in allergic disease prevalence (2, 3). Recent studies underscore the involvement of the epithelial barrier in the pathogenesis of allergic diseases. In 2017, Pothoven and Schleimer introduced the epithelial barrier hypothesis for type 2 inflammatory diseases, positing that dysfunction in the epithelial barrier could catalyze the development of allergic diseases (4). Akdis subsequently expanded upon this hypothesis, suggesting that alterations in the environment and lifestyle resulting from industrialization and urbanization disrupt the epithelial barrier across the skin, upper and lower respiratory tracts, and intestinal mucosa. Epithelial barrier compromise leads to microbial dysregulation, bacterial translocation between epithelial and subepithelial regions, and the onset of tissue microinflammation. Epithelial barrier impairment not only forms the foundation for allergic and autoimmune diseases but also underlies a spectrum of conditions that elicit immunoinflammatory responses to bacteria, viruses, and opportunistic pathogens (5). Current research categorizes diseases marked by compromised epithelial barriers into three main types: 1) chronic conditions stemming from localized barrier defects, resulting in histopathological changes in affected skin and mucous membranes, such as allergic diseases and inflammatory bowel disease (6–8); 2) chronic autoimmune and metabolic diseases, including diabetes, multiple sclerosis, autoimmune hepatitis, etc. (9–12); and 3) Neurodegenerative diseases linked to intestinal barrier deficits and microbial translocations, such as Parkinson’s disease and Alzheimer’s disease (13, 14).

The epithelial barrier hypothesis introduces a novel avenue for investigating the prevention and treatment of allergic diseases. Despite the emphasis on the role of barrier dysfunction in the pathogenesis of allergic diseases, numerous questions persist regarding the mechanisms underlying the disruption of normal barrier function. Therefore, the objective of this review is to furnish a comprehensive overview of the epithelial barrier’s role in allergic diseases, encompassing influencing factors, evaluation techniques, and repair methodologies. By doing so, it aims to pave the way for exploring innovative therapeutic strategies for allergic diseases.

## 2 Epithelial barrier dysfunction and allergic diseases

### 2.1 The epithelial barrier

#### 2.1.1 Composition of the epithelial barrier

**Figure 1:**
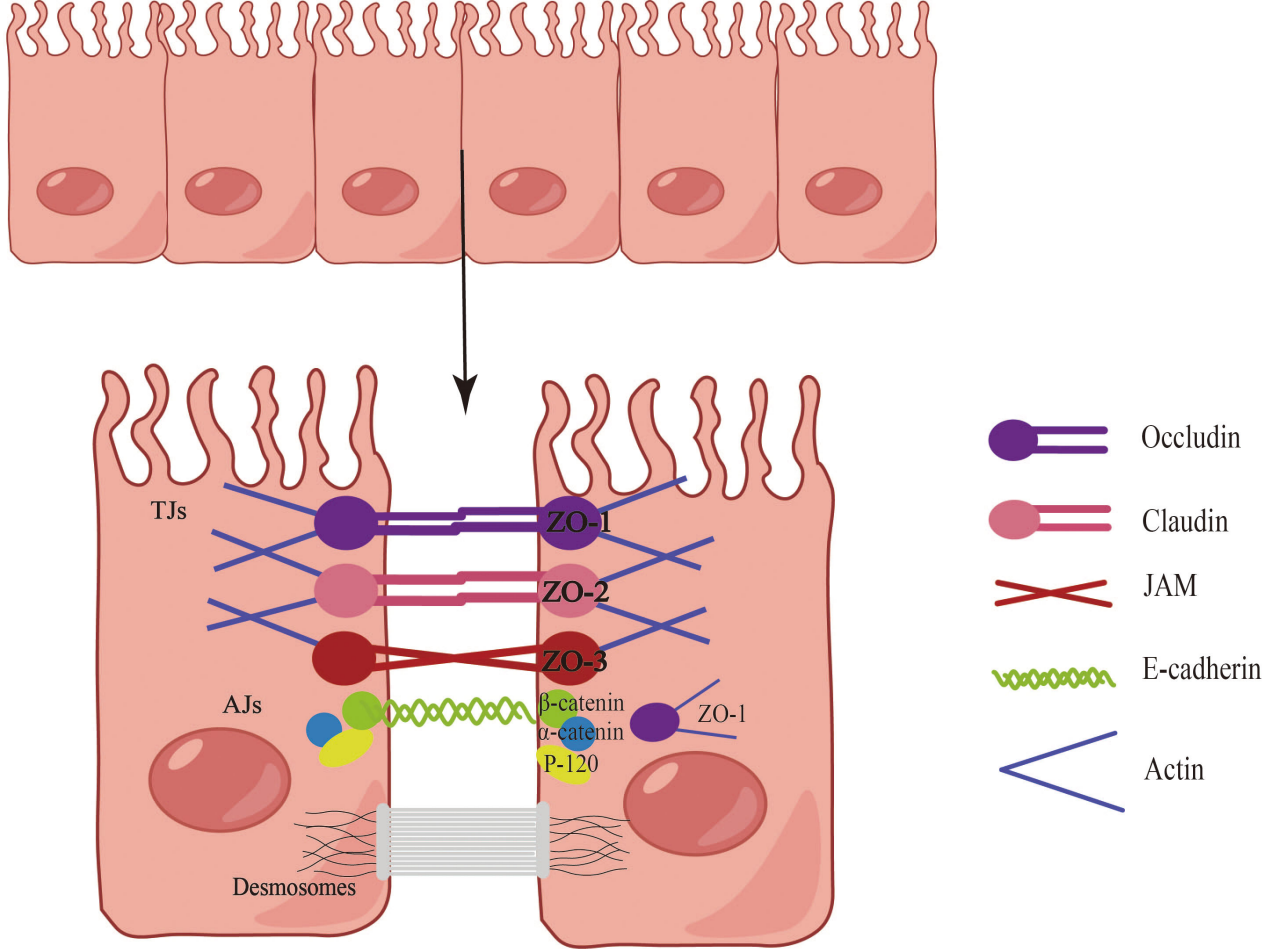
Composition of the epithelial barrier. Epithelial cells establish barriers through the intricate interplay of tight junctions (TJs), adherens junctions (AJs), and desmosomes. The TJ complex is comprised of claudins, occludins, and junctional adhesion molecules, with major cytoplasmic proteins including ZO-1, ZO-2, and ZO-3. AJs, positioned directly below the TJ, encompass E-cadherin, actin, vinculin, α-catenin, and β-catenin. Desmosomes, characterized by a symmetrical structure involving two adjacent plasma membranes, play a vital role in establishing and maintaining stable cellular junctions.

The epithelial barriers of the skin, gastrointestinal system, and respiratory tract exhibit both similarities and differences in terms of structure, function, and biochemical properties. For instance, the skin provides a robust physical barrier, while the epithelial ciliary movement in the airways continuously clears particulate matter, facilitating gas exchange. In the gastrointestinal system, the epithelium facilitates a broad range of nutrient and water exchange while concurrently offering protection against microbes, toxins, and environmental exposures (15). Consequently, epithelial tissues serve multifaceted roles encompassing physical, chemical, and immune barrier functions. Notably, the epithelial cells at each site collaboratively form a cohesive epithelial barrier through cellular connections (**Figure 1**). These connections are established by tight junctions (TJs), adherens junctions (AJs), and desmosome (16). AJs consist of diverse components such as E-cadherin, actinin, vinculin, α-catenin, and β-catenin, while TJs form a complex involving claudins, occludins, and junctional adhesion molecules (17). TJs create a barrier by sealing the apical boundary, preventing the unhindered entry of microorganisms, toxins, and pollutants, and regulating the paracellular transport of ions and certain small molecules (18). AJs play a crucial role in initiating and maintaining intercellular adhesion, contributing to the establishment and regulation of the apicobasolateral membrane structure (18). Desmosomes, characterized by a symmetrical structure comprising two adjacent plasma membranes separated by a 30-nanometer cellular gap, possess a central dense layer or midline flanked by mirrored triple-electron dense plaques (19). Desmosomes play a pivotal role in establishing and maintaining stable cell junctions.

#### 2.1.2 Epithelial barrier and type 2 immune response

**Figure 2:**
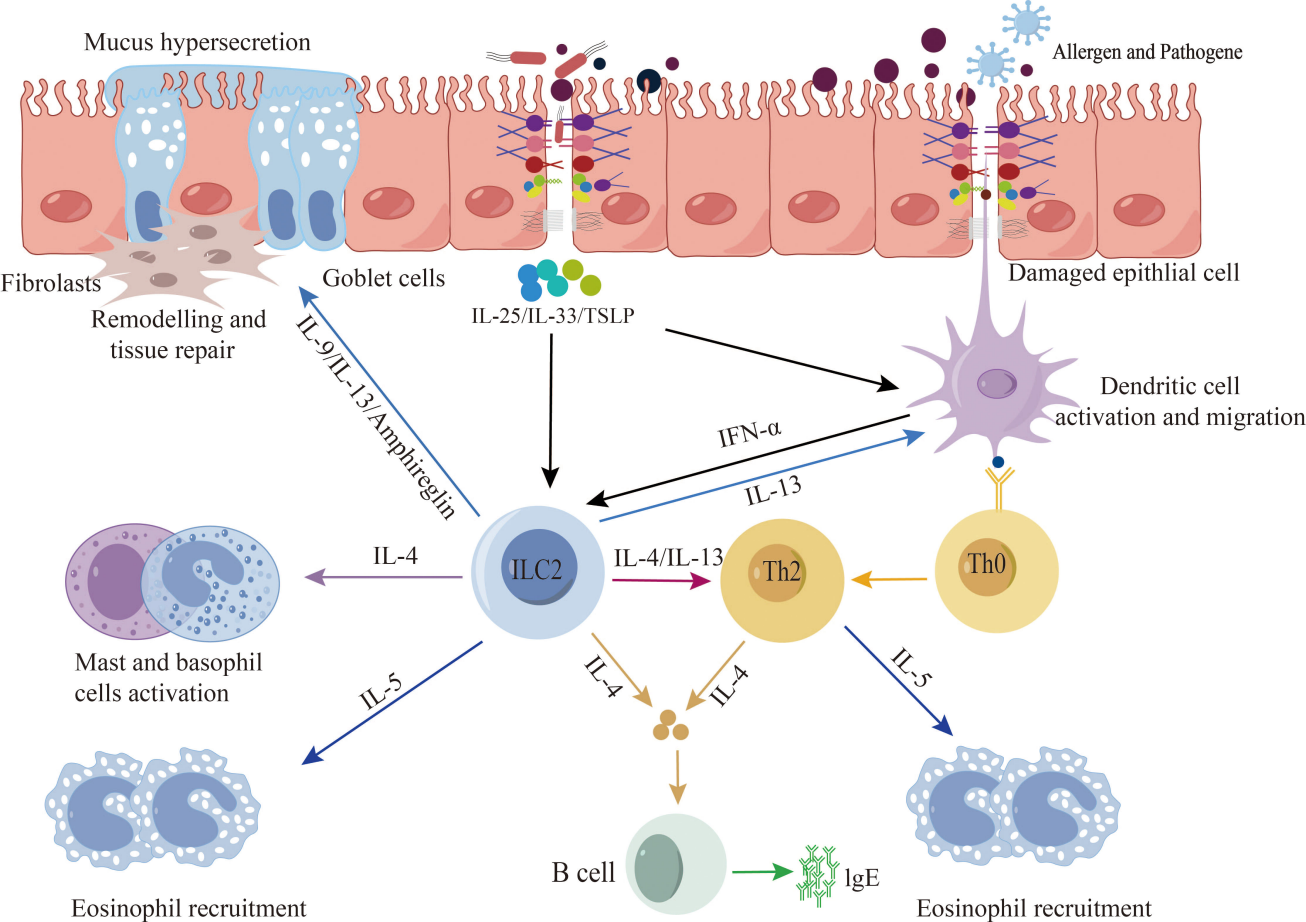
Epithelial barrier and type 2 immune response. When exposed to allergens, pathogenic bacteria, pollutants, etc., a compromised epithelial barrier undergoes activation, leading to the release of alarm factors by epithelial cells, including interleukin-25 (IL-25), IL-33, and thymic stromal lymphopoietin (TSLP). Additionally, activation may occur through the stimulation of dendritic cells, subsequently presenting antigens to Th2 cells and ILC2s cells. This activation process promotes the release of type 2 cytokines, thereby triggering and exacerbating type 2 immune responses.

Disruption of the epithelial barrier emerges as a common pathway contributing to the initiation and exacerbation of allergic diseases (20, 21). Research has unequivocally established the involvement of the epithelial barrier in the pathological progression of allergic diseases (5). Dysfunction in the epithelial barrier across various human tissues is characterized by cell differentiation, compromised junction integrity, and impaired innate defenses. Genetic predisposition, environmental influences, and aberrant inflammation collectively promote the dysfunction and breakdown of the epithelial barrier, thereby precipitating the onset and progression of allergic diseases (22). Studies indicate that compromised epithelial barriers activate epithelial cells, leading to the release of alarm factors such as interleukin-25 (IL-25), IL-33, and thymic stromal lymphopoietin (TSLP). This, in turn, promotes cytokine release by type 2 innate immune cells, triggering and exacerbating type 2 immune responses (23, 24). Epithelial cells play a pivotal role in the initiation and exacerbation of allergic diseases. Key cytokines, including IL-4, IL-13, IL-5, IL-9, TSLP, and IL-33, feature prominently in allergic diseases, as they stimulate the development of inflammatory responses and tissue repair (25–27). However, inflammatory cytokines such as IL-4, IL-3, IFN-γ, and TNF-α have been demonstrated to disrupt epithelial barrier function through various mechanisms. In primary bronchial epithelial cells (PBEC) cultured at the gas-liquid interface, IFN-γ and TNF-α collaboratively impair barrier function, leading to decreased expression of ZO-1 and JAM (28). IL-4 and IL-13 induce epithelial barrier dysfunction by inhibiting the expression of ZO-1, occludin, E-cadherin, and β-catenin (**Figure 2**) (29).

### 2.2 Epithelial barrier dysfunction and allergic diseases

#### 2.2.1 Epithelial barrier dysfunction and asthma

Asthma, characterized as a chronic inflammatory disease of the airways, heavily relies on the integrity of respiratory epithelial cells, which collectively establish a physical, functional, and immune barrier. This barrier is crucial in safeguarding the host from potential hazards associated with inhaled environmental risk factors, thereby maintaining the overall health of the host. Prolonged exposure to external stimuli, however, can lead to the manifestation of epithelial barrier dysfunction within the asthma phenotype (30). The human airway epithelium is composed of predominant ciliated epithelial cells, mucous-secreting goblet cells, club cells, and airway basal cells (6). In a healthy airway, TJs and AJs establish connections between epithelial cells, forming a closed barrier that effectively prevents the invasion of risk factors. This cell barrier is naturally coated with mucus containing antimicrobial agents, peptides, and antibodies, serving as a protective measure against the intrusion of pathogenic bacteria and allergens (17, 31). However, patients with asthma often exhibit airway epithelial barrier disorders characterized by an increase in basal and goblet cells and a decrease in terminally differentiated ciliated cell (32), This is frequently accompanied by basement membrane thickening and epithelial exfoliation, resulting in the formation of Creola bodies composed of exfoliated epithelial clusters (4). Research indicates that during the course of asthma pathology, the number of MUC5B decreases, while the expression of MUC5AC increases, leading to mucus overproduction, airway obstruction, and the accumulation of mucus materials, ultimately inducing chronic inflammation (33). In addition, studies highlight the disruption of tight junctions and adherent junctions in the epithelial barrier as typical features of asthma. Similarly, deficiencies and dysfunction of E-cadherin, α-catenin, ZO-1, and occludin have been identified in patients with asthma (34), resulting in impaired barrier function. Moreover, a damaged airway epithelial barrier activates cytokines (IL-4, IL-5, and IL-13) released by ILC2s, further exacerbating the damage to the epithelial barrier (20, 35). Beyond the damage to epithelial barrier function caused by inflammation in asthma, several recent studies indicate the potential existence of barrier function abnormalities in airway epithelial cells in asthma patients (36). Further in-depth research is still needed to elucidate the mechanism of action of the epithelial barrier during asthma pathogenesis.

In summary, sustained exposure to risk factors such as allergens, pathogenic bacteria, and viruses, coupled with immune inflammatory responses, can lead to severe damage to epithelial barrier components. This damage results in the disruption of epithelial barrier function and structure, ultimately increasing barrier permeability and contributing to the onset and exacerbation of asthma.

#### 2.2.2 Epithelial barrier dysfunction and AR

AR, characterized as an inflammatory condition affecting the nasal mucosa and mediated by allergens, exhibits an escalating incidence, particularly in industrialized and modernized regions, influenced by diverse factors. Recent investigations have brought attention to the role of nasal mucosal epithelium in the pathogenesis of AR (6). The nasal epithelium, acting as the primary interface for allergens, pollutants, and pathogens, anatomically comprises two segments: the initial third of the nasal cavity features stratified squamous epithelium, while the remaining two-thirds possess a pseudostratified columnar ciliary epithelium with goblet cells covering the basement membrane (17). In a healthy state, coordinated ciliary movement and mucus secretion constitute the primary defense mechanism, preventing compromise of the nasal mucosal barrier by various risk or irritant factors. However, in the pathological context of allergic rhinitis, the central role played by the dysfunction and disruption of TJs becomes evident, compromising the integrity of the nasal epithelial barrier and subsequently hindering ciliary motility and mucus secretion (37, 38). Under normal conditions, nasal epithelial cells are bound together by TJs, forming a complex known as an occlusion zone (ZO), inclusive of claudin, occludin, and junction adhesion molecules (37). Notably, studies have revealed diminished expression and immunoreactivity of E-cadherin and ZO-1 in the nasal epithelium of individuals with allergic rhinitis (39). Furthermore, Protease-activated receptor-2 (PAR-2) has been implicated in diminishing the expression of ZO-1 and claudin-1, with the latter involved in epithelial barrier dysfunction in AR (40). Subsequent *in vitro* assessments of barrier function using primary epithelial cells from AR patients demonstrated diminished barrier function and reduced expression of tight junction proteins occludin and ZO-1 in comparison to healthy controls (41). Additionally, type 2 cytokines (IL-4, IL-5, IL-13, TNF-α, etc.) produced during immune-inflammatory responses can directly impact the nasal epithelial barrier, resulting in decreased expression of ZO-1 and E-cadherin (28, 39). Furthermore, proteases from certain allergens have been demonstrated to disrupt epithelial TJs and induce AR disease (4).

In summary, current studies suggest a pervasive disruption of epithelial barrier function and increased permeability in AR patients. This accelerates the penetration of allergens through the nasal epithelial barrier, contributing to the onset and development of AR. Multiple pathways in the pathogenic process of AR lead to damage to the epithelial barrier, necessitating further in-depth research to comprehensively understand the underlying mechanisms.

#### 2.2.3 Epithelial barrier dysfunction and AD

AD stands as the most prevalent chronic inflammatory skin disease influenced by a combination of genetic and environmental factors (42, 43). Current insights into AD pathogenesis encompass skin barrier dysfunction, epigenetic alterations, immune factors, skin, and intestinal dysbiosis, alongside interactions with external risk factors (2, 44, 45). The pathology of AD is distinguished by a disruption of skin barrier function. The human epidermis comprises the stratum basale (SB), stratum spinosum (SS), stratum granulosum (SG), and stratum corneum (SC) (43). The SC, situated as the outermost layer above keratinocytes and Langerhans cells (LC), plays a pivotal role in skin barrier function (46). Studies reveal that the SC consists of proteins such as filaggrin, involucrin, loricin, and an outer lipid layer. Particularly, filaggrin, encoded by the FLG gene, serves as the primary source of natural moisturizing factor (NMF) in SC (47). Mutations in FLG can significantly reduce NMF in AD patients, closely correlating with disease severity and emphasizing NMF reduction as a common feature in AD (47, 48). Currently, FLG gene mutations resulting in epithelial dysfunction stand as the most prevalent and severe genetic risk factor for AD (49). Notably, epithelial injury can trigger innate immune responses, activating the release of pro-inflammatory cytokines and chemokines by keratinocytes, and enhancing antigen presentation by Langerhans cells and dermal dendritic cells. Even in subjects with FLG null-mutation, type 2 immune inflammation can lead to decreased FLG expression in AD-afflicted skin (50–52). Additionally, FLG mutations elevate skin pH and activate skin proteases, inducing skin barrier dysfunction (53). Additionally, FLG mutations elevate skin pH and activate skin proteases, inducing skin barrier dysfunction (54). Jungersted et al. have indicated that the pathogenesis of AD extends beyond FLG mutations (55). During epidermal differentiation, keratinocytes sequentially alter gene expression programs, culminating in terminal differentiation and the formation of a mature corneal layer. In AD-afflicted skin, the loss of corneodesmosin disrupts barrier lipids, impairing skin barrier and antimicrobial function (56). Furthermore, AD alters other proteins in the corneal layer, including corneal adhesion, loricrin (LOR), and involucrin (IVL), resulting in compromised skin barrier function and type 2 immune response (56–59). Additionally, lipids such as ceramides are reduced, and the release of inflammatory cytokines during allergic reactions can further decrease the lipid content of SC, exacerbating barrier dysfunction in AD patients (60–62). Various environmental factors contribute to the disruption of skin barrier function, including detergents, microplastics, particulate matter (PM), etc., confirmed to alter the integrity of the skin barrier (51). Specifically, SC tight junctions can be disrupted by anionic surfactants in commercial detergents, while other components can release inflammatory cytokines that aggravate impairment of barrier function (1, 63). Furthermore, dysregulation of the skin microbiome exacerbates damage to the skin’s barrier function (51, 64, 65). Analyses of bacterial communities in AD states reveal that Staphylococcus aureus colonization contributes to the pathogenesis and aggravation of AD, with intestinal microbiota dysbiosis also considered a significant factor in AD pathogenesis (1, 66). According to existing research, AD can progress from skin diseases to food allergies, allergic rhinitis, and later asthma, a phenomenon commonly referred to as the atopic march (43, 67, 68).

In summary, the skin structure is compromised by a range of environmental and genetic factors, including FLG mutations, skin microbiota colonization, intestinal microbial dysbiosis, and environmental pollution hazards. Skin barrier dysfunction allows exposure to allergens, bacteria, and fungi, triggering immune responses and leading to the onset of AD. Moreover, the progression of AD can further induce the development of allergic diseases such as allergic rhinitis and food allergy.

#### 2.2.4 Epithelial barrier dysfunction and FA

FA represents an immune-inflammatory-mediated adverse reaction to specific foods (69, 70). The underlying mechanism across various food allergies involves immune activation and the disruption of tolerance to the intake of specific foods. Dysfunctions of the gastrointestinal tract and skin barriers can contribute to food sensitivities (71). Notably, CD103^+^ DCs mediate immune tolerance in the gastrointestinal tract, while CD11b^+^ dermal DCs and Langerhans cells (LCs) mediate cutaneous tolerance (72, 73). In the pathogenic process of food allergy, impaired development of regulatory T (Treg) cells is observed, being replaced by the production of helper T2 (Th2) cells. These Th2 cells drive IgE conversion and the expansion of allergic effector cells, thereby exacerbating the immune response associated with food allergy (72). The intestinal epithelial barrier is not a static physical barrier but maintains a dynamic balance with the intestinal microbiome and immune cells (74). Comprising a single layer of columnar epithelium interspersed with goblet cells, encased by a layer of mucus, the intestinal barrier protects the host from penetration by digestive enzymes and microorganisms (67). Intestinal epithelial cells selectively allow the absorption of nutrients, electrolytes, and water while defending against the invasion of harmful microorganisms and toxins (75). The mucus layer, the outermost part of the intestinal epithelium, effectively blocks harmful substances and tissues (74). It consists of an outermost mucus rich in antimicrobial peptides and immunoglobulin A, and an inner layer of mucus adhering directly to neighboring epithelial cells (74, 76). The inner layer of mucus, primarily composed of glycocalyx produced by goblet cells, prevents antigens from invading the lamina propria of the intestinal mucosa (74, 77). Moreover, studies have highlighted the role of TJs as a key component in controlling intestinal barrier permeability in epithelial cell junctions (76). In the context of FA, impaired function of the intestinal epithelial intercellular junctions leads to barrier dysfunction and loss of osmotic function. This results in increased permeability, allowing allergens to penetrate the intestinal barrier and stimulate the submucosal immune system. Damaged epithelial cells release inflammatory cytokines, such as TSLP, IL-25, and IL-33, inducing cutaneous DCs and other cell types to shift the immune response from tolerance to hypersensitivity. This process is involved in allergic sensitization and triggering reactions by activating innate lymphoid cells type 2 (ILC2) and producing IL-4 and IL-13 (69).

In summary, when stimulated by allergens, epithelial cells release alarmins, activating immune cells and antigen-presenting cells. This promotes the occurrence of immune responses and the release of cytokines and inflammatory mediators, further exacerbating the destruction of the epithelial barrier. This, in turn, increases the permeability of the intestinal barrier, forming a vicious circle and aggravating the degree of food allergy disease (78).

#### 2.2.5 Microbial dysbiosis and the epithelial barrier in allergic diseases

With the advancement of industrialization, modernization, and urbanization, various factors such as environmental pollution, increased consumption of processed foods, reduced contact with animals, and excessive hygiene practices have contributed to a rising incidence of allergic diseases. Changes in the environment, health, and lifestyles have significantly impacted microbial diversity and homeostasis. Recent research indicates that alterations in the gut, oral, and skin microbiota may trigger immune-mediated diseases, including autoimmunity, allergies, and chronic inflammatory conditions (79). Host-microbiota interactions play a fundamental role in immune system development (79). The local immune system not only adapts to the presence of host-beneficial microbiota, maintaining internal homeostasis, but also responds appropriately to pathogenic microorganisms (79). Microbial dysbiosis, a key focus in the field of epithelial barrier dysfunction (5, 80). refers to the phenomenon where changes and loss of biodiversity result in an unstable microbial ecosystem dominated by one or several microorganisms. This alteration disrupts the immune balance maintained by the gut, skin, and respiratory microbiota, leading to disease development (81, 82). Beneficial microbiota on mucosal surfaces regulating various aspects of barrier homeostasis, including barrier permeability, TJ expression, angiogenesis, local microinflammation, and mucosal tolerance (2). However, microbial dysregulation and epithelial barrier leakage can disturb immune homeostasis. When the epithelial barrier is compromised, the microbiota within the epithelium (beneficial flora, viruses, conditionally pathogenic bacteria, etc.) can migrate between affected epithelial cells or transfer to other sites (11, 83, 84). The occurrence and development of allergic diseases are closely linked to the imbalance of microbial flora in the epithelium. For instance, studies have demonstrated an association between microbiota dysbiosis and asthma (85). Multiple risk factors contribute to asthma development, with dysregulation of external microorganisms and host microbiota playing a significant role (86). Host microorganisms participate in asthma pathogenesis through the metabolites of gut microbes, such as *Lachnospira*, *Veillonella, Faecalibacterium*, and *Rothia (* 87). The “gut-lung axis” proposes that metabolites produced by gut-derived microbiota reach the lungs, shifting the Th2-Treg balance to Treg, protecting against asthma development (79). Similarly, the composition and function of upper respiratory tract microbiota influence asthma pathogenesis, with variations observed at different ages (88). Microbial flora dysbiosis is also implicated in allergic rhinitis (AR). Studies have noted changes in nasal mucosal microbiota associated with AR, including increased abundance of *Staphylococcus aureus, Propionibacterium, Corynebacterium*, and *Bacteroidetes*, and decreased abundance of *Prevotella* and *Streptococcus (* 89). Environmental microbiota exposure in early life may be biologically linked to allergic manifestations, emphasizing the importance of microbial interactions in AR pathogenesis (90). The skin, as the body’s largest organ, is in constant contact with the external environment and harbors a variety of microorganisms between epithelial cells. Disruption of skin function increases skin leakage, allowing resident bacteria and opportunistic pathogens to enter epithelial tissue, inducing inflammatory responses or exerting protective effects. Studies suggest that decreased microbiome diversity in AD patients is associated with increased disease severity and colonization of pathogenic bacteria such as *Staphylococcus aureus*. Topical application of commensal organisms can potentially reduce AD severity (51, 91). The gastrointestinal tract, closely interacting with environmental microorganisms through food ingestion and excretion, is rich in microbiota crucial for maintaining body microenvironment homeostasis. However, damage to the intestinal epithelial barrier increases mucosal permeability, allowing the invasion of pathogenic bacteria and causing diseases (92). Dynamic changes in gut microbiota are implicated in the development of FA (93), with age and diet influencing intestinal flora abundance and FA occurrence (94).

In summary, the interaction between dysbiosis of the microbiota and epithelial barrier dysfunction can contribute to the occurrence and progression of allergic diseases. Microbial dysbiosis alters the microenvironment balance maintained by microorganisms in respiratory tract, skin, and intestinal tissues, leading to increased epithelial barrier permeability, heightened sensitivity to allergens, and the onset and exacerbation of various allergic diseases.

## 3 Factors that cause epithelial barrier damage

With the ongoing processes of modernization, urbanization, and industrialization, various factors have emerged as potential inducers and exacerbators of allergic diseases. These include climate change, environmental pollution, dietary habits, and biodiversity loss, among others. Exposure to these factors can lead to alterations in the structure of the epithelial barrier, impacting the development of allergic diseases by disrupting normal immunomodulation. Numerous studies highlight the prevalent role of impaired epithelial barrier function in the pathogenesis of allergic diseases, emphasizing that the function of the epithelial barrier influences both innate and adaptive immune responses. Currently, a wealth of research has identified several risk factors contributing to epithelial barrier damage in the contemporary environment (18, 95), and detailed information is shown in **Table 1**.

**Table 1:** Environment factors and epithelial barrier.

Exposure factors	Mechanism	Reference
**Particulate matter**	1.Degradation of TJ proteins, including occludin, cladin-1 and ZO-1. 2.Inhibits E-cadherin levels. 3.Induces and increases ROS in epithelial cells. 4.Foxp3 methylation is increased. 5.Increased permeability of lysosomal membranes, oxidative stress, and lipid peroxidation. 6.Causes DNA damage, protein carbonation, and loss of cytokeratin and filaggrin in epidermal structural proteins.	(96–106)
**Nanoparticles**	1.Disruption of the stability of lysosomal membranes, triggering cell death. 2.Induces immature neurotrophic factor imbalance overexpression, leading to apoptosis of lung epithelial cells. 3.Stimulates the release of pro-inflammatory cytokines, including IL-1a, IL-1b, IL-6, TNF-a, and IL-8. 4.Increases the paracellular permeability of human intestinal epithelial cells.	(107–111)
**Ozone**	1.Induce inflammatory cell invasion, resulting in airway inflammation, peribronchial collagen deposition, and airway hyperresponsiveness. 2.Induce oxidative stress, leading to cell stress, desquamation, and cell death. 3.Induce the production of IL−1α and IL-33 by epithelial cells and bone marrow cells. 4.Direct damage to the epithelial barrier.	(112–117)
**Tobacco and e-cigarettes**	1.Increases the permeability of the alveolar epithelial barrier. 2.Lead to decreased gene and protein expression levels for a variety of TJ and AJ proteins. 3.Disruption of intercellular connections and the integrity of the barrier.	(118–121)
**Enzymes in allergens**	1.Cleavage of occlusin to break down TJ and induce intracellular ZO-1 proteolysis. 2.Destroy the tight junctions between epithelial cells, activate protease-activated receptor-2, and produce TSLP. 3.Directly activate the immune system. 4.Disruption of transmembrane adhesion proteins increases epithelial permeability. 5.Induce airway epithelial cells to produce IL-6, IL-8 and MCP-1; Disrupts epithelial tight junctions and induces cell desquamation.	(122–127)
**Micro And nanoplastics**	1.Change the folding of proteins, change their secondary structure. 2.Interact with lipid bilayers and alter cell membranes. 3.Penetrates the gastrointestinal barrier and induces intestinal dysbiosis. 4.Causes oxidative stress, leading to autophagy in human lung epithelial cells.	(128–133)
**Detergents**	1.Disruption of the integrity of the epithelial barrier. 2.Increase transepidermal water loss (TEWL) from the stratum corneum of the skin. 3.Damage to TJs and related molecules. 4.Induce the release of IL−25, IL−33 and TSLP. 5.Change microbial homeostasis and disrupt the interaction between mucus and bacteria.	(63, 134–136)
**Bacteria**	1.Decreased the expression of occlusin and ZO-1 proteins. 2.Secreted α-hemolysin, which interacts with ADAM10 and degrades E-cadherin protein. 3.Reduces the levels of alveolar occludin, ZO-1, claudin-5, and VE-cadherin. 4.Disrupts epithelial integrity and increases permeability. 5.Disrupts the host actin cytoskeleton and impairs cell-cell adhesion.	(137–141)
**Viruses**	1.Resulting in the deletion of ZO-1 in the tight junction complex. 2.Induce ROS and dsRNA production. 3.Leads to the destruction of TJs and AJs. 4.Increases claudin-2 expression and decreases ZO-1, occludin and claudin-1 protein expression.	(142–146)

### 3.1 Environment factors and the epithelial barrier

#### 3.1.1 Particulate matter

Particulate matter (PM), also known as dust, is a variety of solid or liquid particles that are uniformly dispersed in an aerosol system. PM can be divided into primary particulate matter and secondary particulate matter. Primary particulate matter is particulate matter that is released into the atmosphere from a direct source of pollution, such as combustion soot, automobile exhaust, etc. Secondary particles are formed by some polluting gas components in the atmosphere, or these components are formed by photochemical oxidation reactions with normal components in the atmosphere. According to its aerodynamic diameter, it can be mainly divided into three types (PM0.1, with a diameter of < 0.1μm; PM 2.5, diameter< 2.5 μm; PM10 has a diameter of <10 μm) and due to its small size and toxicity, it is easy to cause diseases, especially respiratory diseases (147, 148). PM can significantly alter epithelial barrier structure. Studies have shown that PM may disrupt the integrity of the airway epithelial barrier by degrading TJ proteins such as occludin, claudin-1, and ZO-1 (96–99). In addition, in mouse models, exposure to PM2.5 inhibits E-cadherin levels in lung tissue, increasing IFN-γ, IL-2, IL-4, IL-6, and IL-10 in bronchoalveolar lavage fluid (100). Likewise, at lower combustion temperatures, aromatic compounds chemically adhere to the surface of metal oxide-containing PMs, resulting in the formation of surface-stable environmentally persistent free radicals (EPFRs). The reactive oxygen species (ROS) are produced by the EPFR redox cycle containing PM, and the antioxidant and inflammatory responses triggered by ROS after inhalation are closely related to the degradation of claudin-1 and occludin proteins in the airway epithelium and mouse lung tissues induced by PM (97, 101). Likewise, at lower combustion temperatures, aromatic compounds chemically adhere to the surface of metal oxide-containing PMs, resulting in the formation of surface-stable environmentally persistent free radicals (EPFRs). The ROS are produced by the EPFR redox cycle containing PM, and the antioxidant and inflammatory responses triggered by ROS after inhalation are closely related to the degradation of claudin-1 and occludin proteins in the airway epithelium and mouse lung tissues induced by PM (102). Furthermore, PM2.5 and PM0.1 can cause increased lysosomal membrane permeability, oxidative stress and lipid peroxidation at low doses, and epithelial cell necrosis at high doses (103). Recent studies have shown that PM can lead to DNA damage, protein carbonation, and loss of cytokeratin and filaggrin in epidermal structural proteins (104–106).

#### 3.1.2 Nanoparticles

Nanoparticles (NPs), also known as ultrafine particles, typically range in size between 1 and 100 nm. Over the past few decades, the prevalence of nanoparticles in environmental pollution has been steadily increasing, contributing significantly to air pollution. Common nanoparticles include titanium dioxide (TiO2) and silicon dioxide (SiO2), frequently utilized as additives in various products such as food, cosmetics, paints, and catalysts. Research has demonstrated that nanoparticles exhibit a high affinity for lipids, leading to the encapsulation and disruption of phospholipid membranes. This disruptive action extends to various lipid-rich environments, including pulmonary surfactants and endothelial cell junctions in lung blood vessels. Additionally, nanoparticles have been shown to compromise the stability of lysosomal membranes, triggering cell death (107). Specifically, TiO2-NP has been implicated in enhancing the synthesis of interleukin-1a (IL-1a) by inducing an unbalanced overexpression of immature neurotrophic factors. This process, mediated through p75NTR signaling, is associated with apoptosis of epithelial cells (108). Needle-like TiO2-NP has also been found to stimulate the release of pro-inflammatory cytokines such as IL-1a, IL-1b, IL-6, TNF-a, and IL-8, disrupting cellular connections and compromising the skin barrier (109). Moreover, nanoparticles tend to accumulate on the surface of intestinal epithelial cells, M cells, and Peyer patches. Through endocytosis by M cells or increased cell membrane integrity, nanoparticles can disrupt the epithelial barrier, leading to cell leakage and increased intestinal epithelial permeability (110). Similarly, NPs have been observed to raise human intestinal epithelial paracellular permeability (111).

#### 3.1.3 Ozone

Ozone, an oxygen allotrope (O_3_), exhibits robust oxidizing properties and finds applications as a bleach, air purifier, and disinfectant. It serves as an alternative to catalytic oxidants in chemical production and is utilized as a rocket fuel oxidizer in its liquid form. Despite its beneficial uses, ozone poses a threat to human health as a harmful air pollutant. Recent studies have revealed that prolonged ozone exposure can lead to various respiratory issues, including bronchial hyperresponsiveness, asthma, chronic obstructive pulmonary disease, pulmonary fibrosis, and, in severe cases, death (112). Acute exposure to ozone is known to impair epithelial barrier function, inducing airway inflammation, peribronchial collagen deposition, and airway hyperreactivity (AHR) (113). This acute exposure manifests as asthma symptoms, reduced lung function, increased inflammatory cell infiltration, and heightened AHR (114). Notably, ozone exposure detrimentally affects key molecules involved in epithelial barrier function (149). The inflammatory response induced by ozone in human lungs is reliant on the production of ROS (115), leading to acute epithelial barrier injury and subsequent disruption (116). Upon acute ozone stimulation, cell stress, desquamation, and death occur through reactive ROS. This process is followed by sustained injury and death of bronchiolar epithelial cells, resulting in protein leakage, infiltration of neutrophils and macrophages, and the production of IL-1a and IL-33 by epithelial and myeloid cells (117). Emerging data indicate that, under the control of the IL-33/ST2 axis, ozone directly instigates barrier damage prior to the inflammatory damage mediated by myeloid cells (117).

#### 3.1.4 Tobacco and e-cigarettes

Tobacco stands out as one of the most prevalent toxic substances in the environment, containing approximately 5,000 chemicals notorious for their toxicity to the respiratory system. Research conducted by Burns et al. has elucidated that exposure to cigarette smoke induces an elevation in the permeability of the alveolar epithelial barrier (118). Furthermore, the enduring exposure to tobacco not only leads to diminished gene and protein expression levels of various TJs and AJs proteins but also culminates in the disruption of intercellular junctions (119, 120). In recent years, e-cigarettes have gained prominence, initially marketed as aids for smoking cessation. Despite their advertised potential for assisting in quitting smoking, there is insufficient evidence supporting this claim. E-cigarettes release evaporated nicotine and flavoring, both of which harbor numerous known toxicities capable of causing damage to the epithelial barrier (121).

#### 3.1.5 Enzymes in allergens that disrupt the epithelial barrier

Enzymes present in allergens serve as enhancers of allergic reactions (122). Numerous studies have provided further validation to the notion that the protease activity inherent in allergens, including molds, pollen, cockroaches, house dust mites (HDMs), and certain foods, plays a crucial role in disrupting epithelial integrity and triggering innate immune responses (150). For instance, investigations have demonstrated that mite allergens contribute to the breakdown of TJs by cleaving occludin, leading to intracellular ZO-1 proteolysis and the consequent disruption of the epithelial barrier (123, 124). Matsumura et al. propose that allergen-derived proteases induce TJs disruption among airway epithelial cells, activate protease-activated receptor-2, and generate TSLP, thereby activating the immune response (122). Additionally, Vinhas et al. identified high-molecular-weight proteases with serine and/or aminopeptidase activity in various sensitized pollens, such as Olea europaea, Dactylis glomerata, Cupressus sempervirens, and Pinus sylvestris. These proteases were found to increase Calu-3 transepithelial permeability by disrupting transmembrane adhesion proteins, including occludin, claudin-1, and E-cadherin (125). Aspergillus, known for its abundant production of proteases, induces the release of cytokines like IL-6, IL-8, and MCP-1 in airway epithelial cells. These enzymes also disrupt tight epithelial junctions and prompt cell desquamation (126). Furthermore, Grozdanovic et al. posit that cysteine proteases in food may contribute to the sensitization process of food allergies by disrupting tight junctions (127). In summary, the enzymes present in various active allergens play a pivotal role in disrupting epithelial barrier function, resulting in heightened epithelial barrier permeability. This, in turn, facilitates the invasion of allergens, triggers immune responses, and contributes to the development of allergic diseases.

#### 3.1.6 Micro and nanoplastics

Microplastics (MPs) are water-insoluble polymer particles with a size of less than 5 mm, while nanoplastic particles have a diameter ranging from 1 nm to 1 μm. Recognized as emerging international pollutants due to their small particle size, microplastics are derived from petroleum and are extensively utilized in various aspects of daily life, presenting potential risks to human health (151). The minute dimensions of micro and nanoplastics facilitate their penetration into tissues, allowing interactions with cells and cellular structural molecules (151). Prolonged exposure to these particles has been associated with various degrees of damage to human health (152). Research by Hollóczki et al. has demonstrated the propensity of nanoplastics to interact with proteins, fundamentally altering the secondary structure of these biological macromolecules critical to their function (128). Molecular dynamics simulations have revealed that polyethylene nanoparticles dissolve into an incoherent single polymer chain network within the hydrophobic core of the lipid bilayer, inducing structural and dynamic changes that impact the cell membrane (129). Recent studies evaluating the effects of polystyrene nanoparticles, with diameters of 25 nm and 70 nm, on human alveolar epithelial A549 cell lines indicated that the toxicological effects of PS-NPs on alveolar epithelial cells were dependent on exposure time, diameter, and concentration (130). Jin et al. further observed that polystyrene MPs could induce dysbiosis in intestinal microbiota, leading to intestinal barrier dysfunction and metabolic disorders in mice (131). Additionally, compared with spherical fluorescent polystyrene (PS) particles of different sizes, particles with a diameter of less than 1.5 nm could directly penetrate the gastrointestinal barrier, causing impaired intestinal function (132). Similarly, nanomaterials have been linked to oxidative stress, inducing autophagy in human lung epithelial cells (133). Amidst rapid development, the escalating use of microplastics and nanoplastics raises serious concerns regarding environmental pollution, with concomitant hidden health hazards for human beings.

### 3.2 Industrial products and epithelial barriers

#### 3.2.1 Detergents

The rise of modernization and industrialization has reinforced people’s emphasis on hygiene, particularly highlighted during the outbreak of the new coronavirus pneumonia epidemic. This attention to hygiene has led to widespread use of detergents and sanitizing products. However, excessive contact and utilization of these cleaning agents can contribute to various diseases, especially skin and respiratory conditions. Research indicates that detergents can play a role in triggering the development of allergic diseases by influencing epithelial barrier disorders (20, 153). Even at minimal concentrations, anionic surfactants and detergents have been shown to directly compromise the integrity of cutaneous keratinocytes and bronchial epithelial barriers by disrupting TJs and associated molecules (63, 134). Long-term use of cleansers, as demonstrated by Douwes et al., can elevate transepidermal water loss (TEWL) in the stratum corneum of the skin, subsequently increasing the risk of contact dermatitis (135). The heightened risk of airway inflammation is linked to the interaction of airway epithelium with environmental air pollutants. Residues of detergents on recently cleaned clothing and floor surfaces can be easily inhaled into the airways, impairing airway barrier function and bronchial epithelial cells. Studies have revealed that emulsifiers can thicken mucosal surface fluid, entrap commensal bacteria, disrupt healthy interactions between epithelial cells and commensal bacteria, alter the microbiota, and interfere with mucosa-bacterial interactions, thereby inducing intestinal inflammation (136). Current research is ongoing to determine whether detergent residues from cleaned dishes can damage the esophageal or gastrointestinal epithelial barrier. Identification of such effects would necessitate the implementation of appropriate avoidance measures (20).

### 3.3 Pathogen and epithelial barriers

#### 3.3.1 Bacteria

In the pathological mechanisms of epithelial barrier dysfunction, dysbiosis of the microbiota has been reported (5, 154). Among them, bacteria were significantly associated with epithelial barrier dysfunction. Bacterium infections mainly rely on its ability to disrupt the epithelial barrier by affecting cell-cell junctions and altering the expression of TJ and AJ proteins. For example, *Staphylococcus aureus* colonization and release of enterotoxin B (SEB) can reduce epithelial cell integrity and increase mucosal permeability by decreasing the expression of occlusin and the expression of ZO-1 protein during ALI culture of polyp epithelial cells (137). Similarly, the addition of purified *Staphylococcus aureu*s V8 protease to ALI-cultured human nasal epithelial cells (HNECs) also found the same barrier integrity impairment and discontinuous expression of ZO-1 (155). Studies also have shown that *Staphylococcus aureus* secreted α-hemolysin interacts with the metalloproteinase domain-containing protein 10 (ADAM10), which can lead to the cleavage of AJ protein E-cadherin and disrupt the lung epithelial barrier in mice (138). Furthermore, Peter et al., when studying human lung tissue with *Streptococcus pneumoniae* infection, found that pneumococcal infection reduced the levels of alveolar occludin, ZO-1, claudin-5, and VE-cadherin (139). In dextran sulfate sodium (DSS)-induced mouse models of colitis and Caco-2 cell lines, *Fusobacterium nucleatum* was found to disrupt epithelial integrity and increase permeability by regulating the expression and distribution of tight junction proteins ZO-1 and occulin, causing an aberrant inflammatory response and aggravating colonic inflammation (140). Besides, bacteria can also cause dysfunction of the epithelial barrier through other pathways. Studies have shown that Pseudomonas aeruginosa mediates the disruption of the epithelial barrier by using four effectors: protein exosomes (Exo) S, ExoT, ExoU and ExoY (141). For example, ExoS and ExoT disrupt the host actin cytoskeleton and induce barrier disruption by impairing cell-to-cell adhesion, where ExoY disrupts barrier integrity without cytotoxicity, while another effector protein, ExoU, produces rapid necrotic cytotoxicity (141). In the human body, beneficial commensal bacteria are able to protect and maintain the homeostatic balance of the microenvironment in the body, and the destruction of the epithelial barrier function by pathogenic bacteria leads to an increase in its permeability, which not only enables pathogenic bacteria to reach the subepithelial tissue, but also promotes the secondary invasion of other allergens and pathogens.

#### 3.3.2 Viruses

Viruses are a non-cellular organism that is tiny, simple in structure, contains only one nucleic acid (DNA or RNA), and must parasitize and replicate in living cells. Some studies have found that epithelial dysfunction has a certain correlation with the invasion of viruses. Sajjan et al. found the loss of ZO-1 in the tight junction complex of rhinovirus (RV)-infected cells in a mouse model, and pointed out that RV promotes bacterial binding and translocation by disrupting airway epithelial barrier function, leading to disease occurrence (142). It is worth noting that RV can stimulate the production of ROS and destroy the epithelial barrier, and can also disrupt the epithelial barrier function through dsRNA produced during RV replication (143). In addition, The E protein of SARS coronaviruses and novel coronaviruses interacts with PALS1, a tight junction-related protein, and alter tight junction formation and epithelial morphology, which is manifested by the loss of PALS1 leading to the destruction of TJs and AJs (144, 145). Similarly, Smallcombe et al. found that the molecular composition of TJs in the Respiratory syncytial virus (RSV)-infected airways was altered, as evidenced by significant upregulation of claudin-2 expression and downregulation of ZO-1, occludin, and claudin-1 protein expression, suggesting that increased claudin-2 expression contributes to airway epithelial barrier leakage (146). Different viruses have different mechanisms of epithelial barrier damage, but all of them can increase epithelial barrier permeability and lead to disease infection. Therefore, strategies to maintain barrier function have great potential in the development of antiviral drugs.

## 4 Evaluation and restoration of epithelial barriers

### 4.1 Evaluation of epithelial barrier function

Currently, various methods are employed to assess epithelial barrier function, encompassing the measurement of biomarkers, epithelial barrier permeability assays, tissue biopsy, and epithelial cytology, as detailed in **Table 2**. Transepidermal water loss (TEWL) has emerged as a widely utilized tool for evaluating epithelial barrier dysfunction (156). This process involves a sensor making contact with the skin surface to measure the amount of moisture evaporating from the skin. TEWL measurements, when used in conjunction with stratum corneum stripping (STS), offer insights into the integrity of the skin barrier (43). Tissue biopsy and specific analyses of epithelial cytology serve as additional approaches to assess cell junction structures and associated protein status (156). Ongoing research explores innovative methods for analyzing the skin barrier, including minimally invasive and scarless STS analysis combined with proteomics (43). Mass spectrometry-based protein analysis has revealed significantly lower expression levels of proteins linked to the skin barrier (filaggrin-2, corneodesmosin, desmoglein-1, desmocollin-1, and transglutaminase-3) and natural moisturizing factors (arginase-1, caspase-14, and gamma-glutamyl cyclotransferase) at lesion sites in patients with atopic dermatitis, both with and without a history of herpes eczema (157). Evaluation of gut barrier permeability employs various chemicals, such as sugar, polyethylene glycol, and ^51^Cr-EDTA, directly assessing barrier function (158). Indirect assessments of mucosal integrity through potential blood biomarkers are also employed. Clara cell protein (CC16) serves as a potential biomarker for airway epithelial injury, with increased serum CC16 observed in acute or chronic lung disorders characterized by elevated airways permeability (159). Strategies for indirect mucosal integrity assessment involve detecting molecules present in the blood, normally found in the intestinal lumen (e.g., LPS), or identifying elevated levels of proteins constituting the intestinal barrier (intestinal fatty-acid binding protein (I-FABP) or tight-junction molecules). These markers indicate intestinal wall damage or elevated concentrations of signaling barrier regulators, such as zonulin, in the blood (158). Moreover, the gold standard for evaluating epithelial barrier function at present involves an assessment combining electrophysiological measurements and probes of varying molecular sizes (158). Rinaldi et al. conducted direct *in vivo* assessments of epidermal barrier function using electrical impedance (EI) spectroscopy, emphasizing the rapid and reliable diagnostic capabilities of electrical impedance spectroscopy in detecting skin barrier defects (160).

**Table 2:** Methods for assessing the epithelial barrier.

Methods	Inspection and analysis	Reference
**TEWL**	Measure the amount of water lost from the body through SC diffusion.	(43, 156)
**Tissue biopsy and epithelial cytology-specific analysis**	Minimally invasive and scarless STS analysis combined with proteomics.	(43, 156, 157)
**Epithelial barrier permeability assays**	By tracking the metabolism of markers, the permeability of the epithelium can be judged.	(158)
**Biomarkers**	Testing the blood levels of CC16, connexin, I-FABP, and zonulin.	(158, 159)
**Electrical impedance spectroscopy**	Direct assessment of epidermal barrier function *in vivo*.	(158, 160)

TEWL, Transcutaneous water loss.

CC16, Clara cell protein16.

FABP, intestinal fatty acid-binding protein.

### 4.2 Epithelial based interventions for allergic diseases therapy

Presently, restoring epithelial barrier function is recognized as a novel strategy for the treatment and prevention of allergic diseases. Notably, Hagner et al. discovered reduced adrenomedullin expression in airway epithelial cells of asthma patients, and supplementation with adrenomedullin promoted the repair of airway epithelial damage (161). Pim1 kinase activity has been identified as crucial for maintaining airway epithelial integrity and preventing pro-inflammatory cytokine secretion induced by HDMs (162). Histone deacetylase (HDAC) activity is implicated in allergic inflammation and tight junction dysfunction between epithelial cells. Increased HDAC activity is an underlying mechanism leading to dysregulation of epithelial cell repair. Inhibition of HDAC activity emerges as a potential strategy to restore nasal epithelium integrity, offering a new avenue for allergic rhinitis therapy (163). Moreover, treatments for allergic diseases play a role in repairing epithelial barrier function. Yuan et al. reported that allergen-specific immunotherapy (SIT) reduced airway inflammatory infiltration and hyperresponsiveness in allergic mice, restoring airway epithelial integrity. This treatment also attenuated Der f-induced airway epithelial endoplasmic reticulum (ER) stress and epithelial apoptosis. Importantly, 4-PBA, an ER stress inhibitor, was found to inhibit IL-25-induced apoptosis of airway epithelial cells dependent on PERK activity (164). Research on intestinal and cutaneous epithelial barrier repair has gained attention. Sun et al. demonstrated that AMP-activated protein kinase (AMPK) enhanced intestinal barrier function and epithelial differentiation by promoting the expression of CDX2 (165). Additionally, He et al. highlighted that different concentrations of vitamin A weakened LPS-induced intestinal epithelial permeability, enhanced the expression of tight junction proteins, and improved intestinal barrier function (166). Various approaches exist to prevent and restore skin barrier integrity, encompassing the use of emollients, moisturizers, occlusive agents, environmental control, and more. Emollients have been reported to reduce the incidence of AD by approximately 50%, exhibiting a protective effect on the skin barrier (167, 168). Emollients and moisturizers in AD serve to protect the skin, form a physical barrier, and retain moisture. Environmental factors, such as allergens, temperature and humidity, skin irritants, and PM2.5, can contribute to skin barrier damage. Effective prevention of skin damage involves controlling these environmental factors. Studies indicate that increased colonization of *Staphylococcus aureus* on the skin in AD is linked to the loss of commensal bacteria (169). This implies that skin barrier repair can be achieved through microbiota regulation. Current research suggests that human-derived bacteria can reduce *Staphylococcus aureus* colonization in AD, pointing to the potential of microbiome balance regulation as a therapeutic and preventive approach for allergic diseases (43).

## 5 Conclusion

The epithelial barrier hypothesis has emerged as a promising avenue for disease prevention and treatment. In the context of contemporary developments in modernization, industrialization, and commercialization, various external risk factors, such as environmental pollution, global warming, widespread detergent use, and biodiversity changes, continually stimulate the epithelial barriers in the skin, respiratory tract, and intestines. This persistent stimulation compromises the integrity of the epithelial barrier, leading to dysbiosis, microbiota translocation in interepithelial and subepithelial regions, and the development of tissue microinflammation. Impaired epithelial barrier function increases permeability, elevating the risk of diverse diseases, especially allergic conditions.

The physical barrier of the epithelial barrier acts as a defense against stimuli like allergens and pathogenic bacteria. Breaches in this physical barrier prompt epithelial cells to release alarm signals (IL-25, IL-33, and TSLP) and activate antigen-presenting cells like dendritic cells. Subsequently, ILC2s cells and Th2 cells are activated, initiating a type 2 immune response. Conversely, the secretion of type 2 immune factors can influence epithelial cells, exacerbating or repairing epithelial barrier dysfunction. Consequently, strategies aimed at treating and restoring epithelial barrier function can be integrated into allergic disease treatment approaches, contributing to disease prevention and management. This review highlights several measures to prevent epithelial barrier dysfunction and address allergic diseases: (1) Control of Environmental Factors: Minimize or avoid exposure to risk factors that harm the epithelial barrier. (2) Development and Use of Safe Products: Adopt safe cleaning products, emollients, and other related items. (3) Mastery of Epithelial Barrier Assessment: Detect epithelial barrier damage promptly through effective assessment methods for preventive purposes. (4) Strengthening the Mucosal Barrier: Prevent bacterial translocation, block colonization by opportunistic pathogens, and reinforce the mucosal barrier. Understanding the role of the epithelial barrier in the mechanisms of allergic diseases can pave the way for innovative preventive and therapeutic methods in future research.

## Author contributions

HL: Data curation, Formal analysis, Writing – original draft. YZ: Data curation, Formal analysis, Writing – original draft. LY: Writing – review & editing. QZ: Writing – review & editing. XW: Writing – review & editing. SQ: Funding acquisition, Supervision, Writing – review & editing. BC: Funding acquisition, Methodology, Project administration, Supervision, Writing – review & editing. XZ: Funding acquisition, Project administration, Supervision, Writing – review & editing.
